# Public health research in India in the new millennium: a bibliometric analysis

**DOI:** 10.3402/gha.v8.27576

**Published:** 2015-08-14

**Authors:** Anuska Kalita, Sachin Shinde, Vikram Patel

**Affiliations:** 1 Department of Population Health, IKP Trust, New Delhi, India; 2 Sangath, Goa, India; 3 London School of Hygiene and Tropical Medicine, London, United Kingdom; 4 Public Health Foundation of India, New Delhi, India

**Keywords:** public health research, research capacity, research systems, health research funding, bibliometry, India

## Abstract

**Background:**

Public health research has gained increasing importance in India's national health policy as the country seeks to address the high burden of disease and its inequitable distribution, and embarks on an ambitious agenda towards universalising health care.

**Objective:**

This study aimed at describing the public health research output in India, its focus and distribution, and the actors involved in the research system. It makes recommendations for systematically promoting and strengthening public health research in the country.

**Design:**

The study was a bibliometric analysis of PubMed and IndMed databases for years 2000–2010. The bibliometric data were analysed in terms of biomedical focus based on the Global Burden of Disease, location of research, research institutions, and funding agencies.

**Results:**

A total of 7,893 eligible articles were identified over the 11-year search period. The annual research output increased by 42% between 2000 and 2010. In total, 60.8% of the articles were related to communicable diseases, newborn, maternal, and nutritional causes, comparing favourably with the burden of these causes (39.1%). While the burdens from non-communicable diseases and injuries were 50.2 and 10.7%, respectively, only 31.9 and 7.5% of articles reported research for these conditions. The north-eastern states and the Empowered-Action-Group states of India were the most under-represented for location of research. In total, 67.2% of papers involved international collaborations and 49.2% of these collaborations were with institutions in the UK or USA; 35.4% of the publications involved international funding and 71.2% of funders were located in the UK or USA.

**Conclusions:**

While public health research output in India has increased significantly, there are marked inequities in relation to the burden of disease and the geographic distribution of research. Systematic priority setting, adequate funding, and institutional capacity building are needed to address these inequities.

Although research is increasingly recognised as one of the driving forces behind global health and development, the research output from low- and middle-income countries (LMICs) such as India compares poorly with that of high-income countries (1–5). This phenomenon has been powerfully captured by what the Global Forum for Health Research popularised as the ‘10/90 gap’: the fact that of the over $70 billion spent worldwide on health research each year, only about 10% is invested in research into 90% of the Global Burden of Disease (GBD). This inequity in the global distribution of health research is further compounded by regional inequities, for example, in the biomedical focus of research, and in geographical and population representation. As a result, the knowledge generated by health research does not adequately address the needs of countries and hinders the implementation of evidence-based policy and practice. It is in this context that there are increasing calls for strengthening health research capacity in developing countries as a ‘critical element for achieving health equity’ (6, 7).

The public health research situation in India is characteristic of the low priority to public health more generally. A recent review by Dandona et al. (8) observed that only 3.3% of the 4,876 health research studies published from India during 2002 were devoted to public health. Clearly, public health research in India is grossly under-represented and requires strategic planning, investment, and resource support if there is to be a positive change in the production of such research in the country and, by its application, the promotion of healthier lives for its population (9). A focus on addressing health inequalities, on evidence-based policy making, on universal health care, and achievement of the Millennium Development Goals are notable public health goals of the new millennium, both globally and in India. In India, public health research has been emphasised as a core investment and tool to guide policy and practice as the country embarks on an ambitious agenda to universalise health care (10, 11). The formation of the Department of Health Research is an example of a step by the government in this direction. This is an institution created in 2007 by the Indian government under the Ministry of Health and Family Welfare – which is the central ministry for health in India. The primary mandate of this department is to promote and co-ordinate basic, applied, operational, and clinical research; provide guidance on research governance; promote inter-sectoral and international collaborations; as well as advance training and grants in medical and health research (12).

It is in this context, that we undertook a systematic situational analysis of public health research in India in the new millennium, with the aim of describing public health research output, whether its focus reflects the current burden of diseases, whether the research is equitably distributed in the country, the research institutions, and funders and collaborations for public health research.

## Methods

Bibliometric analysis is a method used to describe patterns of publication within a given field or body of literature (13–15). The methodology used in this study parallels other bibliometric studies undertaken to evaluate research production in specific scientific disciplines and/or world regions (16–18). Two data sources were selected: PubMed, an open-access international database of medical journals and IndMed, an open-access database of Indian medical journals. The search strategy was determined by the operational definitions of relevant terms – public health and public health research – which are the focus of this study. Notably captured by Acheson in 1999 and by Last in 2000, several definitions of public health exist, which typically reflect the wide scope of public health itself (19, 20). Definitions of both public health [as stated by the World Health Organization (WHO) in 1998] and of public health research (stated by the Strengthening Public Health Research in Europe) accept that the key common points are the population approach (public health) and the production of generalisable knowledge (research) (21, 22).

In case of PubMed (www.ncbi.nlm.nih.gov/pubmed), an ‘advanced search’ of the title, keywords, and the entire article was conducted with Medical Subject Headings (MeSH), a comprehensive vocabulary for the purpose of indexing journal articles in the life sciences. In the MeSH tree, health care is a ‘major topic,’ which includes public health as a sub-head (23). Since *health care* also included articles that were not related to public health, a combination of the two MeSH terms were used.

The search terms used were:

1. MeSH major topic – *health care* + *public health*, AND
2. Text word – *India*, *AND*
3. Publication date – from 2000/01/01 to 2010/12/31

The search yield was 7,844 references. Selected abstracts were directly imported into an EndNote library. To ensure that all articles related to public health have been included, analyses to test the accuracy of the search terms were conducted for combinations of MeSH major topic *health care* with MeSH terms *diseases, mental disorders, social sciences*, and *Anthropology*, *Education*, *Sociology*, and *Social Phenomena*. For the first accuracy analyses, it was found that all relevant articles were included in the primary search (*healthcare*+*public health*). For the fourth accuracy analysis, 2,566 articles were found to be relevant to our study but were not included in the original search yield. These were added to make the total PubMed yield 10,410.

IndMed is a database covering peer-reviewed Indian biomedical journals and complements PubMed. It covers 62 journals indexed from the publication year 1985 onwards. After reviewing the ‘advanced search’ option in IndMed with ‘*public health*’ in keywords and the year of publication (individually for each year from 2000 to 2010), we observed that the results were unlikely to be complete. For instance, only 19 abstracts were listed for the year 2000 with this search combination from all journals. Thus, we used a different strategy searching each journal individually. Of the 62 journals, 9 were indexed in PubMed. Of the remaining 53, 17 journals were selected on the basis of table of content analysis revealing at least 5% of the articles per randomly selected set of issues on themes of public health research. The indexing of these 17 journals was incomplete for most journals. To address these gaps, additional searches were conducted. The first strategy involved web-searches of the table of contents from the journal websites (four journals had websites with archives of abstracts). For seven journals, external websites or databases were used to close data gaps. For the remaining six journals, hand searches were conducted in the following libraries – the National Medical Library and the B.B. Dikshit Library at the All India Institute of Medical Sciences, Delhi, and the Dorabji Tata Library at the Tata Institute of Social Sciences, Mumbai.

We screened abstracts of all identified articles from either of these two databases for inclusion for bibliometric analysis. In case of articles that did not have abstracts, the full text was screened. The following inclusion criteria were used:

1. Published in English language.
2. Must be data-based (either primary and/or secondary).
3. Studies must be undertaken in India – either exclusively, or in India as one of the countries in a multi-country/study.

To ensure reliability, two independent reviewers screened each paper and the two EndNote libraries were matched, thus leading to a reliability check of 100% of the selected abstracts. In addition, a randomly selected sample of 500 abstracts from across the 11 years was manually checked by a third reviewer.

Based on the inclusion criteria, 5,869 articles from PubMed and 2,024 articles from IndMed were found to be eligible, yielding a total sample of 7,893 articles. Each abstract (or full-text of papers without abstracts) of the 7,893 eligible papers were reviewed by two independent reviewers and categorised under biomedical disease focused papers or papers that described determinants, policy, and practice. Biomedical disease focused papers were further categorised into three categories based on the GBD Study definitions, viz., GBD 1 included studies on communicable diseases, maternal and neonatal health, and nutritional disorders; GBD 2 included studies on non-communicable diseases and mental and behavioural disorders; and GBD 3 included studies on injuries. Articles that involved research on two or more GBD categories were classified under each of them. The non-disease category included articles on social determinants of health, history of medicine, ethics, policy, and programmatic research that is not related to specific disease burden categories. Abstracts were categorised independently by the two reviewers; discrepancies were addressed by consulting a third reviewer.

To analyse the disease focus and geographical distribution of public health research in India, data were extracted into a spreadsheet for the following parameters from each article 1) disease focus – as per the GBD categories; 2) location of the research study across all states and union territories of India; 3) corresponding author's institution (as a proxy for the research institution leading the study); and 4) location of the corresponding author's institution across all states and union territories of India.

To analyse funding source and international collaborations, we randomly selected 1,600 articles (20% of the total sample) for more detailed analyses of the full manuscript. We also attempted to fill data gaps in any of these categories of information through web-based searches and direct communication with authors. This yielded 1,076 papers with information about collaborations (approximately 67% of the sub-sample, and 13.7% of the total sample), and 870 papers with funding sources (approximately 54% of the sub-sample and 11% of the total sample).

Descriptive analysis and frequencies were used to describe absolute outputs over time, examine outputs in different categories of GBD over time, geographical distribution of research/research institutions, collaborations, and funders.

### Ethics statement

The study was reviewed and has been approved by the Institutional Review Board of Sangath (Sangath-IRB).

## Results

### Absolute research output

**Fig. 1:**
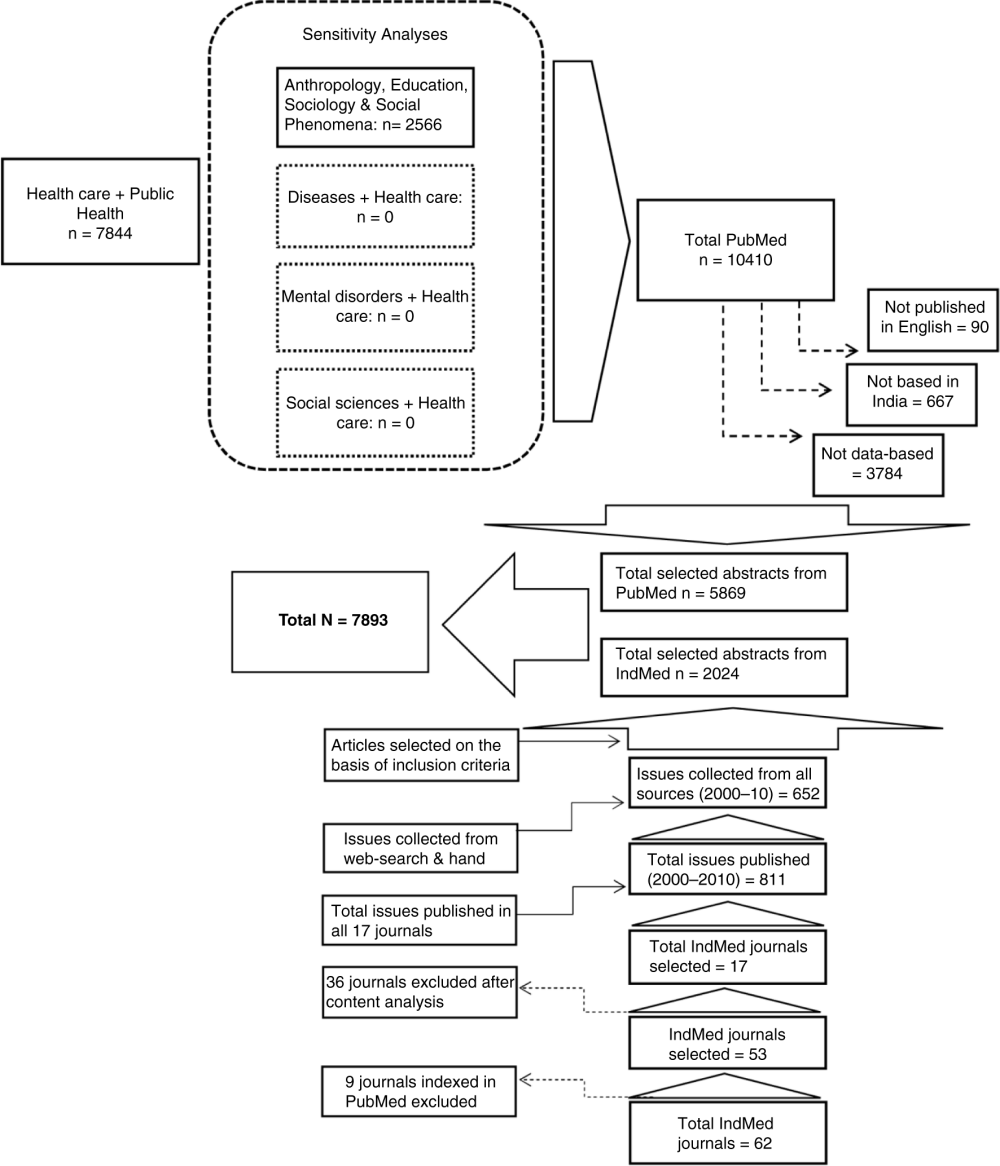
The process of data collection for bibliometric analysis.

**Fig. 2:**
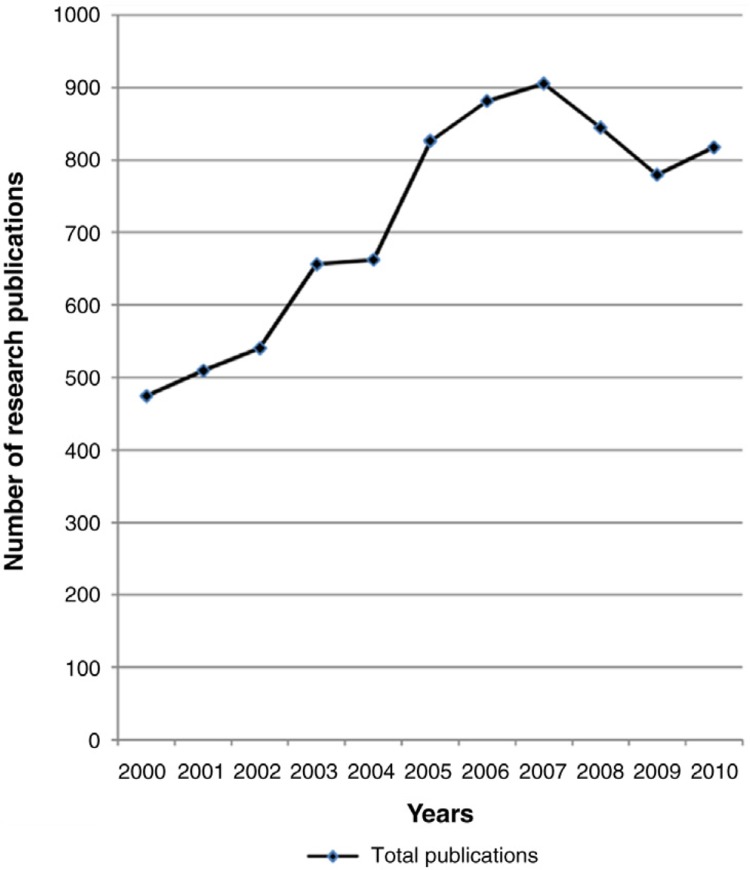
Absolute research output from India during the decade 2000–2010.

The total number of eligible articles included in the bibliometric analysis from both PubMed and IndMed was 7,893 (5,869 from PubMed and 2,024 from IndMed). The process of data collection is shown in Fig. 1. There was a trend of an increase in publication over time, with the total number of publications in 2010 (*n* = 817) showing a 72.3% increase compared with 2000 (*n*=474). Figure 2 shows the trend of published research output over the decade. Although there was an overall increase in the number of publications between 2000 and 2010, the number declined sharply between 2007 and 2009. Specific reasons for this decline were not detected.

### Distribution of public health research

**Fig. 3:**
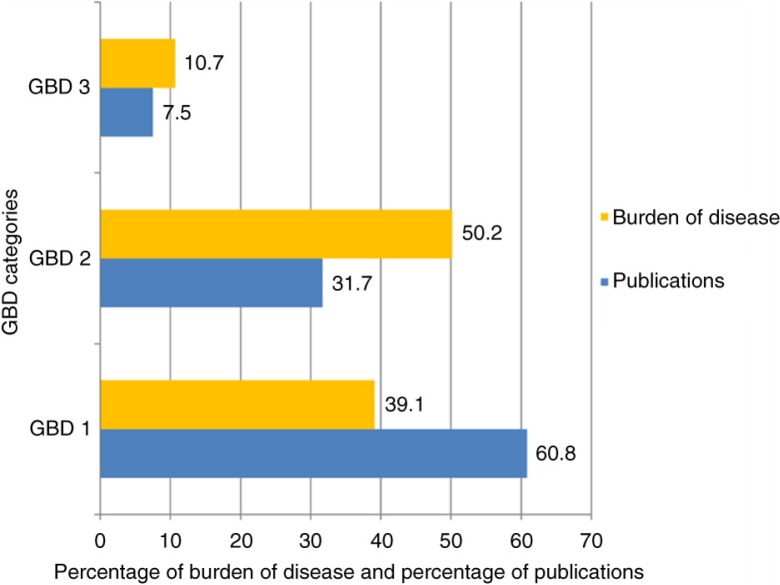
Publication research focus relative to the burden of disease in India during 2000–2010. *Note*: Burden of disease (DALYs) for GBD categories are estimates for the year 2004.

Out of the 7,893 papers, 6,103 reported the topic of research as one or more of the GBD conditions. We observed that the majority of the papers with a biomedical focus were related to conditions in the GBD 1 category across all 11 years (60.8%, 3,711/6,103), compared with a burden of disease, as estimated at the mid-point of the decade in 2004, of 39.1% (Fig. 3). The proportion of lost DALYs (Disability Adjusted Life Years) caused by conditions under GBD 2 category for India was 50.2% in 2004. Compared to this burden, only 31.7% (1,933/6,103) publications focused on conditions under this category. The proportion of research focused on diseases in GBD 3 is 7.5% (458 out of 6,103), which is slightly lower than the burden of disease in this category (10.7%) in India.

**Fig. 4:**
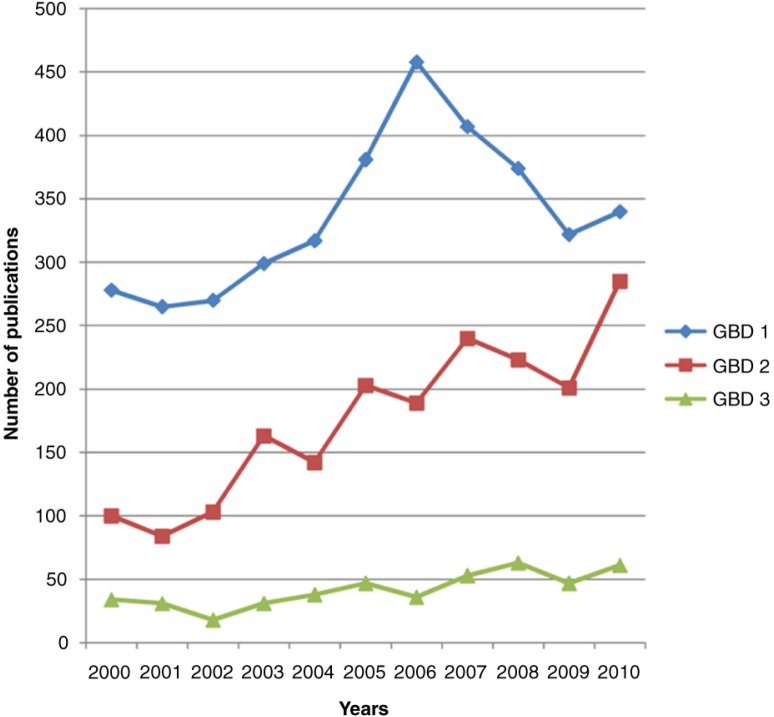
Trends in publications from India by global burden of disease categories from 2000 to 2010.

We observed a trend of reduced proportion of GBD 1 and a proportionate increase in those related to GBD 2 over time, although the proportionate distribution of research in the later years still does not match the burden of disease reported in the GBD 2010 (Fig. 4).

**Fig. 5:**
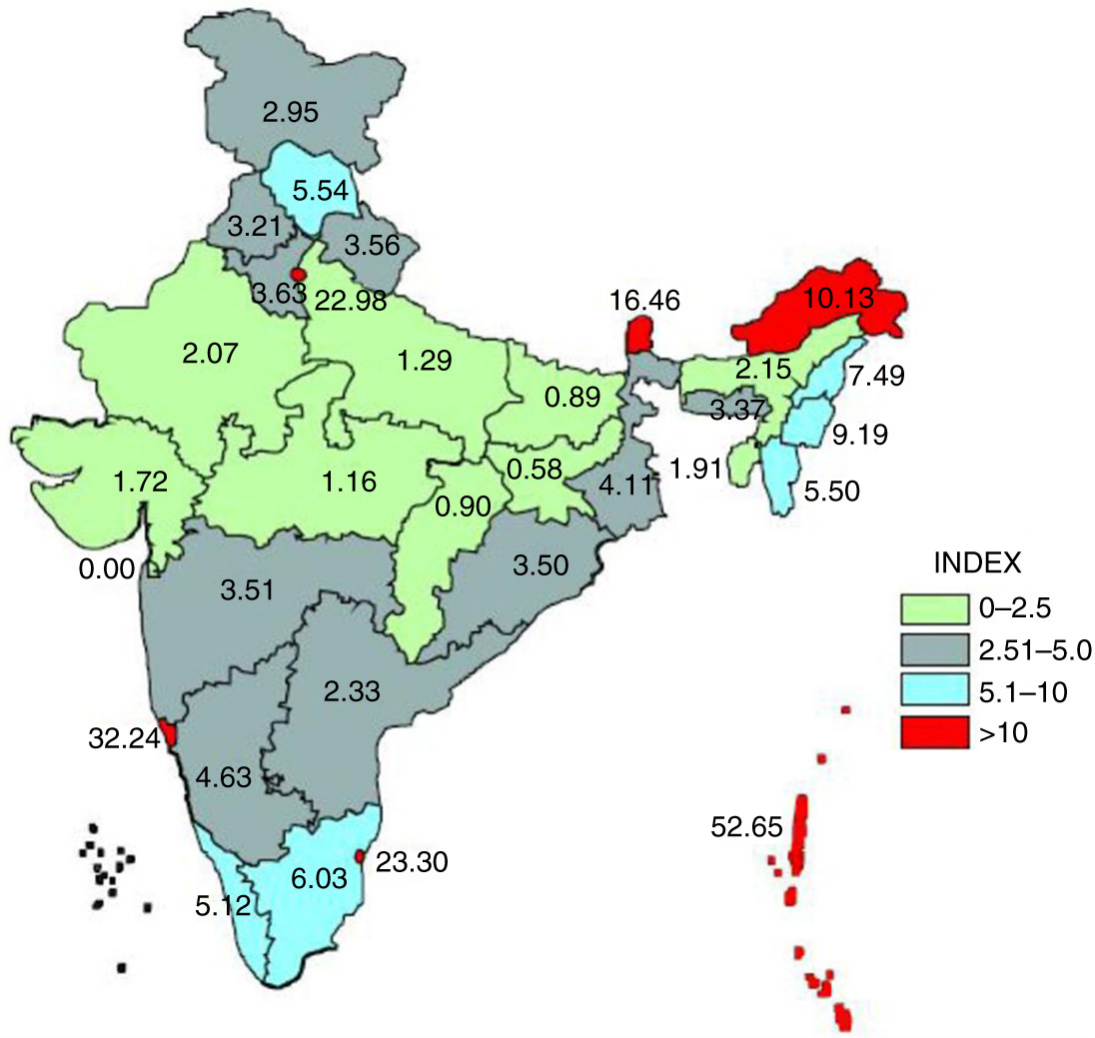
Per capita distribution of research studies in India.

The geographical equity in public health research output is skewed. For this, we considered the Empowered Action Group (EAG) that was constituted by the Ministry of Health and Family Welfare in 2001 to facilitate area-specific interventions for the eight most populous and poorest states (viz. Bihar, Chhattisgarh, Jharkhand, Madhya Pradesh, Rajasthan, Orissa, Uttarakhand and Uttar Pradesh), which together account for 45.9% of India's population and 56.5% of the poor were the location of just 10% of publications (801/7,893) (24). This is presented in Fig. 5.

### The research actors

Out of our total sample of 7,893 papers, 7,706 papers reported corresponding addresses. From this sample, 78.4% (6,044/7,706) reported an Indian research institution. In total, 42.5% (2,572/6,044) of the papers were produced from research institutions located in just three states of Delhi, Maharashtra, and Tamil Nadu. Table 1 lists the 15 leading research institutions in India. Together these institutions produced 21% (1,258/6,044) of the research papers from India during the last decade; the majority of these institutions were located in Delhi and Maharashtra. Another observation was the disparity in production of research even among these top 15 institutions, which ranged from a maximum of 555 papers to a minimum of 13. The north-eastern seven states accounted for the least number of research institutions (1.4%, 111/7,706), while the eight EAG states accounted for 12.7% (979/7,706) of research institutions.

**Table 1:** The 15 leading institutions for public health research in India

Research institution	Location of institution	Number of papers by corresponding author affiliation (out of 6,044)	Percentage of papers
Indian Council of Medical Research	Delhi	555	9.2
All India Institute of Medical Sciences	Delhi	226	3.7
Christian Medical College	Tamil Nadu	147	2.4
Maulana Azad Medical College	Delhi	145	2.0
Post Graduate Institute Medical Education Research	Chandigarh	99	1.6
St. John's National Academy of Health Sciences	Karnataka	44	0.8
Mahatma Gandhi Institute of Medical Sciences	Maharashtra	44	0.7
Jawaharlal Institute of Postgraduate Medical Education and Research	Puducherry	40	0.7
Sree Chitra Tirunal Institute for Medical Sciences and Technology	Kerala	36	0.6
National Institute of Mental Health and Neuro Sciences	Karnataka	30	0.5
Tata Memorial Centre	Maharashtra	27	0.4
King Edward Memorial Hospital	Maharashtra	27	0.4
International Institute for Population Studies	Maharashtra	24	0.3
P.D. Hinduja National Hospital and Medical Research Centre	Maharashtra	17	0.3
Apollo Hospitals	Delhi/Tamil Nadu	16	0.3
Ministry of Health and Family Welfare	Delhi	15	0.2
Vardhman Mahavir Medical College and Safdarjung Hospital	Delhi	15	0.2
Sangath	Goa	13	0.2

Of the 7,706 publications that reported a corresponding author institution, 21.5% (1,662/7,706) were foreign. Based on full-text analyses of the randomly selected sub-sample, a further 19.6% (210/1,076) of papers with first author affiliation to an Indian institution reported foreign collaborators. These 210 papers mentioned a total of 275 different international collaborators. Of the foreign corresponding author institutions, a majority – 65% (1,078/1,662) were from two countries – the United States of America and the United Kingdom. A similar proportion (57.6%) was observed for other foreign collaborators, that is, excluding corresponding author institutions. The leading foreign institutions undertaking public health research in India are shown in Table 2. Together, these institutions led 26.9% (442/1,662) of the papers and were involved in collaborations on 89% (187/210) of the papers.

**Table 2:** The 10 leading international collaborating institutions for public health research in India

International collaborating institution	Location of institution	Number of papers by corresponding author affiliation (*n*=1,662)	Percentage of papers	Number of papers by any author affiliation (with Indian corresponding author) (*n*=210)	Percentage of papers
Johns Hopkins University	United States of America	88	5.3	15	7.1
Harvard University	United States of America	62	3.7	14	6.7
London School of Hygiene and Tropical Medicine	United Kingdom	61	3.7	33	15.7
World Health Organization	Multilateral	54	3.2	30	14.3
University of California	United States of America	42	2.5	16	7.6
University of North Carolina	United States of America	27	1.6	10	4.8
Population Council	United States of America	20	1.2	13	6.2
Centre for Disease Control	United States of America	20	1.2	11	5.2
International Agency for Research on Cancer	France	18	1.1	9	4.3
University of Manitoba	Canada	17	1.0	9	4.3
University of Melbourne	Australia	17	1.0	18	8.6
University College London	United Kingdom	16	0.9	9	4.3

Eight hundred and seventy papers of the sub-sample of 1,600 papers yielded information on funding sources. In total, 34.1% (297/870) listed an Indian funding agency and the remaining two-thirds (573/870) listed a foreign funding source. The main funding institutions supporting public health research in India are listed in Table 3. In total, 81.5% (709/870) of papers were funded by these 10 agencies. While all the four Indian funders are governmental institutions, international funding agencies represent a mix of multilateral and bilateral organisations (WHO and the Department for International Development-UK) and private foundations (Wellcome Trust and the Bill and Melinda Gates Foundation).

**Table 3:** The 10 leading funders of public health research in India

Funding agency	Location of institution	Number of papers (*n*=870)	Percentage of papers
Indian Council for Medical Research	Delhi	98	11.3
Bill and Melinda Gates Foundation	United States of America	93	10.7
World Health Organization	Multilateral	91	10.5
Department of International Funding for Development (DFID)	United Kingdom	86	9.9
Wellcome Trust	United Kingdom	83	9.5
United States Aid (USAID)	United States of America	75	8.6
The World Bank	Multilateral	65	7.4
Department of Science and Technology	Delhi	46	5.2
Ministry of Health and Family Welfare	Delhi	40	4.6
University Grants Commission	Delhi	32	3.7

## Discussion

This paper describes the results of an analysis of public health research in India in the new millennium. The data source was a bibliometric analysis of one of the largest international and the largest national databases of medical research. Our main findings were that while public health research output has increased substantially over the course of the first decade of the new millennium, there is considerable maldistribution of research in terms of the disease focus and the geographical focus. Most research is funded by international donors with relatively low levels of domestic public or private sector investment. International academic partners, particularly from the USA and the UK, play influential roles in research with little evidence of south–south partnerships with other developing countries.

In a country which bears a disproportionate amount of the GBD, it was reassuring to observe that the total number of publications based on public health research in India has substantially increased over the first decade of the millennium; however, this increase (of 72.3%) falls well below that of other middle-income countries such as South Africa (225% increase from 2000 to 2010) (25, 26), Mexico (102% from 1995 to 2004) (27), and Brazil (241% increase from 1995 to 2004) (28). This absolute increase in the volume of publication masks striking inequities both in terms of the research focus and the research settings. Even according to the recent GBD estimates of 2010, while GBD 2 and 3 conditions accounted for 45 and 12% (together 57%) of the burden of disease, just 35 and 7% (42%) of papers focused on these conditions (29). These findings are consistent with the only other bibliometric study from India and those from other LMICs (2–5, 30). This skewed picture has been attributed to the misconceived notion of research agencies and donors regarding the association of these diseases with affluence (27, 31–34) even though the majority of GBD 2 and 3 conditions are more frequent among poorer populations in LMICs (27, 35–40).

In addition to the under-representation of research on leading causes of the burden of disease in India, there is a markedly inequitable representation of vulnerable contexts or population groups in India. Capacities exist, but are unequally distributed, as is evident from the concentration of research institutions in richer states of the country such as Delhi, Maharashtra, West Bengal, and Tamil Nadu. A number of factors contribute to these maldistributions – dependence on foreign funding and donor-driven research priorities, asymmetries in capacities of researchers and institutions leading to a concentration of research in a few subject areas and geographies, and a policy and research-system vacuum. The lack of research institutions in states contributing to the highest proportions of poverty and disease burden in the country potentially contributes to a vicious cycle of low capacity to carry out public health research, which is relevant to these populations.

International institutions, both donors and research partners, play a leading role in public health research in the country. Two-thirds of the publications were based on research funded by foreign donors. This compares unfavourably with other middle-income countries such as Brazil and China where 74.3 and 78.6% of the total health research funding comes from the domestic public sector agencies and only 2.2 and 8.8% comes from international funding agencies (41–44). This reliance on international funding may contribute to the inequities in the distribution of research, such as an undue focus on international goals like the MDGs. These issues of skewed priorities and funding need to be addressed through a significant increase in domestic investments in public health research that is transparent, accountable, and responsive to the burden of disease and the needs of diverse geographical regions and populations of the country. There is also a need for domestic private philanthropies to support public health research; in Brazil, for example, domestic private sector organisations contribute 23.3% investments in public health research (43). Channelling private-sector support towards public health research assumes special relevance in the context of the recent Companies Bill that mandates 2% allocation of profits of listed companies towards corporate social responsibility (45).

Given the inequitable distribution of research institutions and focus areas in the country, the focus of capacity strengthening efforts to build institutions, especially in resource-poor states and in neglected public health focus areas is urgent. However, attracting and retaining researchers within institutions require coordinated strategies that address familiar barriers such as the lack of academic liberty, absence of professional incentives, poor and non-transparent funding, bureaucratic obstacles, and unclear career pathways (9). The weak public health research environment in India needs strengthening through a comprehensive approach. There is often little communication and consultation between the producers of research and the users of research: policy-makers, health providers, civil society, the private sector, other researchers, and the general public. It is important to recognise that the health research process spans the entire spectrum of policies related to knowledge creation as well as its diffusion and use. Therefore, a well-coordinated, systematic approach to health research needs to involve all stakeholders. For instance, priority setting needs to underlie the efforts to increase the quality, relevance, and production of research by considering whether there is a demand for this research. The paucity of forums to interact and share knowledge, inaccessibility of existing global resources and information asymmetry, and the lack of systematic dissemination of research towards policy and practice all lead to a weak research ecosystem.

Collaborations between domestic, as well as international researchers and institutions, can foster such exchange and access. Evidence from South Africa and Brazil suggests that international collaborations dramatically boost the volume of health research publications in high impact peer-reviewed journals (46, 47). To realise the potential of collaborative research, it is crucial that local capacities are strengthened and relationships between domestic and international institutions are based on equal partnerships. An issue of note here is the dominance of the USA and the UK in collaboration for public health research in India. South–south collaborations, either with countries such as Brazil or South Africa with vibrant public health research cultures, or with other countries in South Asia which share similar public health priorities, were negligible. Steps need to be built on to encourage cooperation, such as – facilitating discussions and sharing of national experiences; supporting cross border training; developing networks of researchers, policymakers, and institutions; and increasing political visibility of health (48–50).

The weakness of governance systems that regulate and monitor public health research in the country often lead to insufficient coordination. Research activities in various health-related fields have been fragmented, isolated from each other, and wastefully duplicative. In a context like India, where both financial and human resources are scarce, this is inefficient and sub-optimal. While the Department of Health Research was set up under the Ministry of Health and Family Welfare by the Government of India in 2009–2010 (12), a policy for health research, a clear mandate and empowerment of the Department, and systems of convergence with existing departments and government institutions have yet to clearly articulated. The current need in India is for the health research system to identify priorities, mobilise resources, both public and private, and maximise the use of existing ones, develop and sustain the human and institutional capacity necessary to conduct research, disseminate research results to target audiences, apply research results in policy and practice, and evaluate the impact of research on health outcomes. Good quality research can and must be generated to continuously address critical knowledge and practice gaps to advance innovation in and improve implementation of public health programmes. Such research cannot be viewed as an indulgence in resource-poor states but needs to be at its most creative and relevant in precisely those contexts.

The last decade has seen some positive developments in the area of health. Recommendations for universalisation of health coverage (10) increased investments in health in the 12th Five-Year Plan period (11), and the proposal for a comprehensive and convergent National Health Mission (11) is all desirable goals, which need evidence generation for their effective implementation. Public health research priorities and investments need to be convergent with, and not parallel to, these goals.

This study suffers from the typical limitations of bibliometric analyses, that is, the fact that these miss out on articles or journals, which are not indexed. Another limitation could be the risk of misclassification of articles (in particular regarding focus areas) despite our robust efforts to minimise this bias. Additionally, newer articles published from 2011 till date have not been included within the scope of this study, and we acknowledge that there might be changes in the trends of public health research in India in the last 4 years. Nevertheless, our findings represent the most comprehensive analysis of public health research in India in the current millennium and serve as a reference for the evaluation of future research production metrics.

## Conclusions

While public health research output in India has increased significantly in the first decade of this millennium, there are marked inequities in relation to the burden of disease and the geographic distribution of research. Systematic priority setting, adequate funding, and institutional capacity building are needed to address these inequities. It is imperative that India invests adequately in developing a vibrant and rigorous ecosystem of public health research at the heart of its public health strategy.
